# POLICY SERIES: THE MOVING FORWARD NURSING HOME COALITION: NURSING HOME REFORM AND THE RESIDENTS’ GOALS, PREFERENCES, AND PRIORITIES

**DOI:** 10.1093/geroni/igad104.0960

**Published:** 2023-12-21

**Authors:** Tara McMullen, Tetyana Shippee, Howard Degenholtz

## Abstract

The 2022 National Academies of Sciences, Engineering, and Medicine (NASEM) report called for nursing facilities to provide “care aligned to the individual’s goals and preferences.” Missing has been an efficient process to uncover the individual’s detailed goals, preferences, and priorities (GPPs). GPPs are a care planning process that translates into actionable interventions, supported by quality measurement to determine whether the care provided aligns with those GPPs, and health information technology to collect GPPs efficiently and to determine if the care provided is concordant with the individual’s GPPs. A diverse set of experts representing the Moving Forward Nursing Home Quality Coalition are collaborating to develop an integrated approach to these shortcomings and will discuss these issues in detail including new approaches to the identification and documentation of GPPs for use in a modified care planning process, the status of health information technologies (HIT) processes being developed to support these changes and the development of new quality measures. While the Moving Forward Coalition is focused on nursing facilities, the approaches being developed here to improve care and to standardize the vocabulary for GPPs are applicable to all care settings. Three committees from the Moving Forward Nursing Home Coalition will present work focused on the development of a standardized GPP care planning process to enhance the identification, documentation, communication, and implementation of resident GPPs, the measurement of the care planning process to support process improvement, and the creation of a HIT-enabled process to measure the concordance of care provided with an individual’s GPPs. This is a collaborative symposium between the Aging Workforce, Hospital Elder Life Program, Measurement, Statistics, and Research Design, Systems Research in Long Term-Care, and Technology and Aging Interest Groups.

